# An Enhanced Lightweight IoT-based Authentication Scheme in Cloud Computing Circumstances

**DOI:** 10.3390/s19092098

**Published:** 2019-05-06

**Authors:** Rafael Martínez-Peláez, Homero Toral-Cruz, Jorge R. Parra-Michel, Vicente García, Luis J. Mena, Vanessa G. Félix, Alberto Ochoa-Brust

**Affiliations:** 1 Facultad de Tecnologías de Información, Universidad De La Salle Bajío, Av. Universidad 602, León 37150, Mexico; jrparra@delasalle.edu.mx; 2 Department of Sciences and Engineering, University of Quintana Roo, Blvd Bahía S/N, Chetumal 77019, Mexico; htoral@uqroo.edu.mx; 3 Departamento de Ingeniería Eléctrica y Computación, Universidad Autónoma de Ciudad Juárez, Av. José de Jesús Macías Delgado 18100, Cd. Juárez 32310, Mexico; vicente.jimenez@uacj.mx; 4 Unidad Académica de Computación, Universidad Politécnica de Sinaloa, Ctra. Libre Mazatlán Higueras Km 3, Mazatlán 82199, Mexico; lmena@upsin.edu.mx (L.J.M.); vfelix@upsin.edu.mx (V.G.F.); 5 Facultad de Ingeniería Mecánica y Eléctrica, Universidad de Colima, Av. Universidad 333, Colima 28040, Mexico; aochoa@ucol.mx

**Keywords:** authentication, cloud computing, Internet of Things, mutual authentication, session key agreement

## Abstract

With the rapid deployment of the Internet of Things and cloud computing, it is necessary to enhance authentication protocols to reduce attacks and security vulnerabilities which affect the correct performance of applications. In 2019 a new lightweight IoT-based authentication scheme in cloud computing circumstances was proposed. According to the authors, their protocol is secure and resists very well-known attacks. However, when we evaluated the protocol we found some security vulnerabilities and drawbacks, making the scheme insecure. Therefore, we propose a new version considering login, mutual authentication and key agreement phases to enhance the security. Moreover, we include a sub-phase called evidence of connection attempt which provides proof about the participation of the user and the server. The new scheme achieves the security requirements and resists very well-known attacks, improving previous works. In addition, the performance evaluation demonstrates that the new scheme requires less communication-cost than previous authentication protocols during the registration and login phases.

## 1. Introduction

Information technology has grown rapidly in the past few years, mainly developing new technologies focused on how to take advantage of the Internet. In this sense, innovations such as wireless access, high speed connection, APIs and electronic services have successfully entered the Internet arena. At the same time, researchers and computer professionals have developed new communication technologies, such as Wi-Fi, 4G, 5G, routing protocols, LTE, Bluetooth, RFID, among others, which offer broader ranges to users. In parallel, the cost of technology keeps decreasing, year by year, making Internet connectivity more accessible for everyone through smaller devices as tablets and smartphones.

On the other hand, as the Internet has become ubiquitous, faster, and increasingly accessible to non-technical communities, social networking and collaborative services, emerging technologies such as artificial intelligence, big data, cloud computing, wireless sensor networks and the Internet of Things (IoT) have appeared, enabling people to communicate and share interests in many more ways. As a consequence, these novel technologies are changing the world, again, creating new business opportunities, new applications to enhance safety, comfort, and efficiency reducing human efforts, and new ways to collect and analyse data.

Among these five emerging technologies, the IoT and cloud computing have become more and more relevant to academia and industry. In 1999 Ashton introduced the concept of IoT [1], which is defined as the connection of physical objects (devices/sensors) through the Internet [2]. Cloud computing, on the other hand, was introduced in 1961 by McCarthy [3], and 36 years later, Chellappa explained the concept in the scenario of current technology era [4], defined as a large-scale distributed computing paradigm which drives the economy of many companies based on virtualization, managed computing power, and storage focused on its core business [3].

The IoT applications are classified into the following categories [5,6]: (a) Internet of sensors (IoS), which is a network made up of sensors which collect and transmit a types of data; (b) Internet of energy (IoE), which is a network of smart grids to analyse and control the energy production, consumption, storage, and distribution; (c) machine to machine (M2M) communication is a network of devices/sensors connected through the Internet; and (d) Internet of Vehicles (IoV), which is a network of vehicles that can share information about the state of the road. Another classification is based on the communication models [7]: (a) machine-to-machine communication, which includes multiple devices/sensors connected to exchange data among them without a physical infrastructure; (b) machine-to-cloud communication, which includes devices/sensors that consume services from a cloud server for storing and processing data; and (c) machine-to-gateway communication, which includes devices/sensors that collaborate as a proxy to expand the range of the network.

In the case of cloud computing, we can find four types of deployment models offered by cloud providers. The first type is known as public cloud, where services and systems are available for anyone through an open communication channel. The second type is known as private cloud where services and systems are accessible for certain users/employees or organizations through a secure communication channel. The third type is known as community cloud where the cloud is shared by several organizations. The last type which includes a public and private cloud to share resources is known as hybrid cloud [4].

From these service platforms, cloud computing allows the interconnection of everything around us, including our vital signs, personal and sensitive data, that travel through an open communication channel to different sites [8,9,10]. This is possible because devices/sensors automatically collect a huge amount of data and stores them on cloud servers [11,12,13,14]. However, this also represents a significant disadvantage in terms of security, because large amounts of personal and sensitive data stored in a single database could be accessed without the approval of users [9].

On the other hand, the main advantage of the IoT, ubiquity, is also its main weakness, because it is also necessary to have a high and complex security protocol. According to El-Hajj et al. [6] and Ferrag et al. [5], IoT security requirements include authentication, authorization, integrity, confidentiality, non-repudiation, availability, and privacy to protect the data, nodes, and messages against attacks.

Therefore, to address the security requirements of both emerging technologies is important focus special attention on the authentication process, because it is the first line of defense against potential attackers. The goal of the authentication protocol is verifying the identity of an entity to determine that he/she or device/sensor is who or what it claims to be [5,6]. For this reason, the authentication process is a key component for secure Internet communication.

In this sense, a strong authentication protocol needs to achieve the following two aims: mutual authentication and session key agreement [15]. In addition, the authentication protocol must avoid the denial-of-service, forgery, parallel session, password guessing, replay, smart card loss, and stolen-verifier attacks [15]. In the threat model of any authentication protocol, an adversary or malicious user has the computational power to compute complex operations in low time and control the public communication channel to capture and store any messages. In general, security threats include more than thirty kinds of attacks [5]. However, the most popular attacks used for evaluating authentication protocols are man-in-the-middle, impersonation and forging, and replay attacks [5].

In recent years, authentication protocols have used cryptosystems as countermeasures to enhance security [5]. The main cryptosystems are hash functions, symmetric algorithms (AES), asymmetric algorithms (RSA, D-H, ECC), digital signatures, and ID-based cryptography; and its adoption mainly depends on the deployment, computational power and energy consumption of the device/sensor. Thus, cryptosystems that require more computational power must be implemented in device/sensor that needs high energy consumption. Therefore, we address to lightweight authentication protocols, which require low computational operations. In this way, the studies carried out by Wang et al. in 2015 [16] and 2018 [17] contribute to understanding the security requirements, adversary model, types of schemes, and evaluation criteria of lightweight authentication protocols.

### 1.1. Related Work

In this paper, we refer to lightweight authentication schemes which require low computational operations, such as hash functions and exclusive-OR operation. The first lightweight authentication scheme was proposed by Lamport in 1981 [18]. Lamport introduced the concept of a hash chain to authenticate remote users through an open communication channel. Lamport’s scheme is feasible for practical implementation due to its low computational cost, however, the scheme requires that the server maintains a verification table making it vulnerable to steal personal data. In 1990, Hwang et al. [19] proposed a scheme without a verification table. Later, Lin et al. [20] proposed an authentication scheme based on the asymmetric ElGamal algorithm to improve the security characteristics of Li et al. in [21]. Since 1990, several authentication schemes were proposed to enhance the security of previous ones. These schemes were designed for communication between *n* users and a single server.

Later, Liao et al. [22] proposed an authentication scheme for a multi-server environment. Nevertheless, Liao et al.’s scheme is vulnerable to an insider attack, masquerade attack, server spoofing attack, and registration center spoofing attacks [23]. Hsiang et al. [23] proposed a new authentication scheme to resolve the security drawbacks of Liao et al.’s scheme. However, Martínez-Peláez et al. [24] demonstrated that Hsiang et al.’s scheme is insecure. Two years later, Kim et al. [25] evaluated Martínez-Peláez et al.’s scheme finding security vulnerabilities. The same year, Li et al. [26] evaluated the scheme proposed by Sood et al. [27] finding it insecure. Then, Xue et al. [28] demonstrated the security vulnerabilities of Li et al.’s scheme. Later, Amin et al. [1] proposed an authentication scheme which remedies the security drawbacks of Xue et al.’s scheme. Nonetheless, Challa et al. [29] demonstrated that Amin et al.’s scheme is vulnerable to privileged-insider and impersonation attacks.

In 2019, Zhou et al. [30] proposed a scheme based on hash function and exclusive-or operation to provide authentication on large-scale IoT and cloud computing deployment. They explained that Amin et al.’s scheme cannot resist off-line guessing attacks. They also claimed that their scheme is secure against very well-known attacks.

### 1.2. Contribution

In this work, we review the scheme proposed by Zhou et al. [30] and point out that the scheme has security vulnerabilities and drawbacks which make it insecure. In particular, the scheme is vulnerable to insider, replay and user impersonation attacks. Moreover, the scheme fails to provide mutual authentication and fails to protect secret keys. Therefore, the main contribution of this paper is a new version of the authentication scheme proposed by Zhou et al. which achieves the following security characteristics: mutual authentication and session key agreement. Moreover, our proposal maintains the user’s anonymity against eavesdroppers and requires login phase.

On the other hand, in the security scheme of Zhou et al., neither the server or nor the user knows if the other party is a legal member of the system. For that reason, the scheme includes a new sub-phase called evidence of connection attempt which provides elements for identifying the participants of the authentication request. This sub-phase complements the authentication phase included in [30].

The rest of this paper is organized as follows: Section 2 presents the overview of Zhou et al.’s scheme, in particular registration and authentication phases, and contains the results of the security analysis carried out to the proposal of Zhou et al. based on [5,6,15]. The new proposal is explained in Section 3. Security analysis and performance evaluation are given in Section 4. Conclusions are presented in Section 5.

## 2. Review and Security Analysis of Zhou’s Scheme

Firstly, we provide a brief description about the registration and authentication phases of the scheme proposed by Zhou et al. in [30]. Then, we carry out the security analysis to explain the drawbacks and vulnerabilities found in their scheme.

### 2.1. Registration Phase

This phase is divided in user registration and cloud server registration sub-phases.

#### 2.1.1. User Registration Sub-Phase

In this sub-phase, the user ($U_{i}$) is registered by the control server (*CS*). The communication between $U_{i}$ and *CS* is through a secure channel. The steps involved in this sub-phase are as follows:

Step 1: $U_{i}$ selects an identity $\left(ID_{i}\right)$, pseudo-identity $\left(PID_{i}\right)$, password $\left(PW_{i}\right)$, and nonce $\left(b_{i}\right)$. Then, $U_{i}$ computes $HP_{i}=h\left(PW_{i}∥b_{i}\right)$ and sends the registration request message $M_{1}=\left\{ID_{i},PID_{i}\right\}$ to *CS* were h(·) is a one-way hash function, ∥ represents concatenation operation and ⊕ represents an exclusive-or operation.

Step 2: Upon receiving the registration request message $M_{1}$, *CS* verifies if $ID_{i}$ is valid or not. In case that $ID_{i}$ is invalid, the registration process will be closed. On the other hand, CS computes $C_{1}^{*}=h\left(PID_{i}∥ID_{CS}∥x\right)$ and $C_{2}^{*}=h\left(ID_{i}∥x\right)$. Then, *CS* stores $ID_{i}$ in a database and sends the registration response message $M_{2}=\left\{C_{1},C_{2},ID_{CS}\right\}$ to $U_{i}$.

Step 3: After receiving the registration response message $M_{2}$, $U_{i}$ computes $C_{1}=C_{1}^{*}\;⊕\;HP_{i}$, $C_{2}=C_{2}^{*}\;⊕h\left(ID_{i}∥HP_{i}\right)$ and $C_{3}=b_{i}\;⊕\;h\left(ID_{i}∥PW_{i}\right)$, and stores $\left(C_{1},C_{2},C_{3},PID_{i},ID_{CS}\right)$ in his smart card.

At this point, the user registration sub-phase is over and $U_{i}$ was registered by CS.

#### 2.1.2. Cloud Server Registration Sub-phase

In this sub-phase, the server $\left(S_{j}\right)$ is registered by the control server (CS). The communication between $S_{j}$ and *CS* is through a secure communication channel. The steps involved in this sub-phase are as follows:

Step 1: $S_{j}$ selects an identity $\left(SID_{j}\right)$ and pseudo-identity $\left(PSID_{j}\right)$. Then, $S_{j}$ sends the registration request message $M_{3}=\left\{SID_{j},PSID_{j}\right\}$ to *CS*.

Step 2: Upon receiving the registration request message $M_{3}$, *CS* computes $B_{1}=h\left(PSID_{j}∥ID_{CS}∥x\right)$ and $B_{2}=h\left(SID_{j}∥x\right)$. Then, CS stores $SID_{j}$ and sends the registration response message $M_{4}=\left\{B_{1},B_{2},ID_{CS}\right\}$ to $S_{j}$.

Step 3: After $S_{j}$ receives the registration response message $M_{4}$, $S_{j}$ stores $\left(B_{1},B_{2},SID_{j},PSID_{j},ID_{CS}\right)$.

At this point, the cloud server registration sub-phase is over and $S_{j}$ was registered by CS.

### 2.2. Authentication Phase

**Figure 1:**
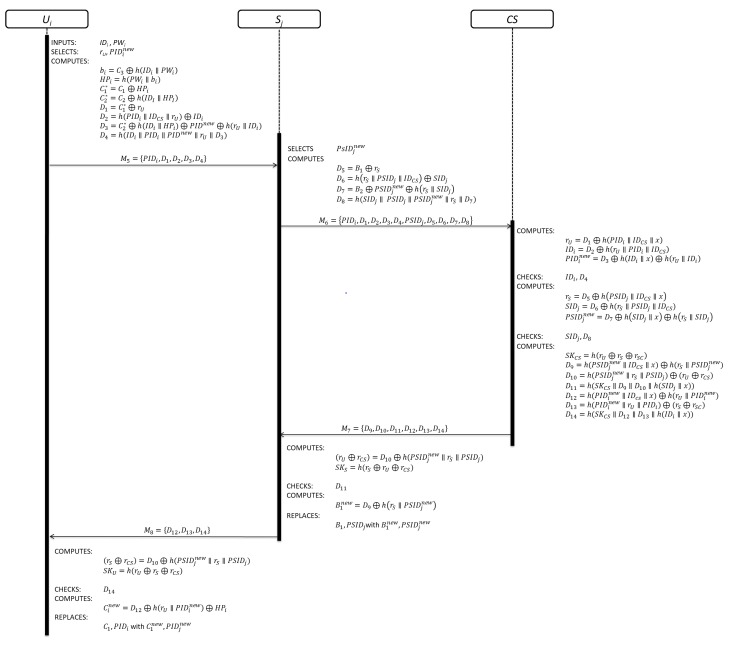
Authentication phase of Zhou et al.’s scheme.

This phase is divided in the following steps and the details are shown in Figure 1:

Step 1: This step is invoked by $U_{i}$, when she/he wants to get access to the service offered by $S_{j}$. $U_{i}$ inserts his/her smart card and keys his/her $ID_{i}$ and $PW_{i}$. Then, the smart card generates a random number $\left(r_{U}\right)$ and new pseudo-identity $\left(PID_{i}^{new}\right)$. After that, $U_{i}$ computes $b_{i}=C_{3}⊕h\left(ID_{i}∥PW_{i}\right)$, $HP_{i}=h\left(PW_{i}∥b_{i}\right)$, $C_{1}^{*}=C_{1}⊕HP_{i}$, $C_{2}^{*}=C_{2}⊕h\left(ID_{I}∥HP_{I}\right)$, $D_{1}=C_{1}^{*}⊕r_{U}$, $D_{2}=h\left(PID_{i}∥ID_{CS}∥r_{U}\right)⊕ID_{i}$, $D_{3}=C_{2}^{*}⊕h\left(ID_{i}∥HP_{i}\right)⊕PID^{new}⊕h\left(r_{U}∥ID_{i}\right)$, and $D_{4}=h\left(ID_{i}∥PID_{i}∥PID_{i}^{new}∥r_{U}∥D_{3}\right)$. Then, $U_{i}$ sends the authentication request message $M_{5}=\left\{PID_{i},\;D_{1},\;D_{2},\;D_{3},D_{4}\right\}$ to $S_{j}$ through an open communication channel.

Step 2: This step is invoked by $S_{j}$. After receiving the authentication request message $M_{5}$, $S_{j}$ selects a new pseudo-identity $\left(PSID_{j}^{new}\right)$ and a random number $\left(r_{S}\right)$. Then, $S_{j}$ computes $D_{5}=B_{1}⊕r_{S}$, $D_{6}=h\left(r_{S}∥PSID_{j}∥ID_{CS}\right)⊕SID_{j}$, $D_{7}=B_{2}⊕PSID_{j}^{new}⊕h\left(r_{S}∥SID_{j}\right)$, and $D_{8}=h\left(SID_{j}∥\;PSID_{j}∥PSID_{j}^{new}∥r_{S}∥D_{7}\right)$. Finally, $S_{j}$ sends the authentication request message $M_{6}=\left\{PID_{i},\;D_{1},\;D_{2},\;D_{3},\;D_{4},\;PSID_{j},\;D_{5},\;D_{6},\;D_{7},\;D_{8}\right\}$ to CS through an open communication channel.

Step 3: This step is invoked by CS. Upon receiving the authentication request message $M_{6}$, CS computes $r_{U}=D_{1}⊕h\left(PID_{i}∥ID_{CS}∥x\right)$, $ID_{i}=D_{2}⊕h\left(r_{U}∥PID_{i}∥ID_{CS}\right)$, and $PID_{i}^{new}=D_{3}⊕h\left(ID_{i}∥x\right)⊕h\left(r_{U}∥ID_{i}\right)$. Then, CS checks if $ID_{i}$ and $D_{4}$ are valid or not. If the verification process fails, CS closes the communication. On the other hand, CS computes $r_{S}=D_{5}⊕h\left(PSID_{j}∥ID_{CS}∥x\right)$, $SID_{j}=D_{6}⊕h\left(r_{S}∥PSID_{j}∥ID_{CS}\right)$, and $PSID_{j}^{new}=D_{7}⊕h\left(SID_{j}∥x\right)⊕h\left(r_{S}∥SID_{j}\right)$. Next, CS checks if $SID_{j}$ and $D_{8}$ are valid or not. If the verification process fails, CS closes the communication. On the other hand, CS selects a random number $\left(r_{CS}\right)$ and computes the session key $SK_{CS}=h\left(r_{U}⊕r_{S}⊕r_{SC}\right)$. Later, CS computes $D_{9}=h\left(PSID_{j}^{new}∥ID_{CS}∥x\right)⊕h\left(r_{S}∥PSID_{j}^{new}\right)$, $D_{10}=h\left(PSID_{j}^{new}∥r_{S}∥PSID_{j}\right)⊕\left(r_{U}⊕r_{CS}\right)$, $=h\left(SK_{CS}∥D_{9}∥D_{10}∥h\left(SID_{j}∥x\right)\right)$, $D_{12}=h\left(PID_{i}^{new}∥ID_{cs}∥x\right)⊕h\left(r_{U}∥PID_{i}^{new}\right)$, $D_{13}=h\left(PID_{i}^{new}∥r_{U}∥PID_{i}\right)⊕\left(r_{S}⊕r_{SC}\right)$, and $D_{14}=h\left(SK_{CS}∥D_{12}∥D_{13}∥h\left(ID_{i}∥x\right)\right)$. Finally, CS sends $M_{7}=\left\{D_{9},D_{10},D_{11},D_{12},D_{13},D_{14}\right\}$ to $S_{j}$ through an open communication channel.

Step 4: This step is invoked by $S_{j}$. Yet receiving the authentication response message $M_{7}$, $S_{j}$ computes $\left(r_{U}⊕r_{CS}\right)=D_{10}⊕h\left(PSID_{j}^{new}∥r_{S}∥PSID_{j}\right)$ and $SK_{S}=h\left(r_{S}⊕r_{U}⊕r_{CS}\right)$. Then, $S_{j}$ checks if $D_{11}$ is correct or not. If the verification process is correct, $S_{j}$ computes $B_{1}^{new}=D_{9}⊕h\left(r_{S}∥PSID_{j}^{new}\right)$ and replaces $B_{1},\;PSID_{j}$ with $B_{1}^{new},\;PSID_{j}^{new}$. Finally, $S_{j}$ sends $M_{8}=\left\{D_{12},D_{13},D_{14}\right\}$ to $U_{i}$ through an open communication channel.

Step 5: This step is invoked by $U_{i}$. After receiving the authentication response message $M_{8}$, $U_{i}$ computes $\left(r_{S}⊕r_{SC}\right)=D_{13}⊕h\left(PID_{i}^{new}∥r_{U}∥PID_{i}\right)$ and $SK_{U}=h\left(r_{U}⊕r_{S}⊕r_{CS}\right)$. Then, $U_{i}$ checks if $D_{14}$. is correct or not. If the verification process is correct, $U_{i}$ computes $C_{i}^{new}=D_{12}⊕h\left(r_{U}∥PID_{i}^{new}\right)⊕HP_{i}$ and replaces $C_{1},\;PID_{i}$ with $C_{1}^{new},\;PID_{i}^{new}$.

### 2.3. Security Vulnerabilities

In this subsection, we explain the weaknesses found in the scheme proposed by Zhou et al. in [30]. The security analysis was conducted based on [5,6,15]. From these works, we performed the security analysis. In this part, we assume the following capabilities of the attacker [31,32]:

- The attacker is a legal member of the system which means that he/she was registered by *CS* and he/she has all the security parameters.
- The attacker can control the public communication channel giving him/her the possibility to intercept, insert, store, delete, or modify any message.
- The attacker has high computational power connected to the public communication channel.

#### 2.3.1. Insider Attack

This attack happens when a malicious user has enough knowledge to attack sensitive data or the whole system. In this scenario, the attacker knows $C_{2}^{*}=h\left(ID_{i}∥x\right)$, computed by CS during the user registration phase. According with Zhou et al., CS uses the same secret key (x) to register users and servers, so he/she can find x from $C_{2}^{*}$, searching exhaustively all possible random number y until $h\left(ID_{i}∥y\right)=C_{2}^{*}=h(ID_{i}∥x$) to know the secret key of CS. This attack is possible because the attacker knows $ID_{i}$ and $C_{2}^{*}$.

#### 2.3.2. Man-in-the-Middle Attack

This attack happens when an attacker has control over the public communication channel providing him/her the possibility to listen the conversation between two entities. Under this scenario, the attacker can intercept the authentication request message $M_{5}=\left\{PID_{i},\;D_{1},\;D_{2},\;D_{3},D_{4}\right\}$ sent from $U_{i}$ to $S_{j}$. Under this situation, the attacker can achieve the following active attacks.

Replay Attack

The attacker can transmit the last authentication request message $M_{5}$ sent to $S_{j}$, at any given time and from any given place, to impersonate a legitimate $U_{i}$. The description of the attack is as follows:

Step 1: The attacker has, at-least, one authentication request message $M_{5}$ sent by $U_{i}$ to $S_{j}$, and the attacker knows $ID_{CS}$ and x.

Step 2: The attacker sends an authentication request message $M_{5}^{replay}$ to $S_{j}$. The message contains the same information transmitted in previous communication.

Step 3: The attacker uses $ID_{CS}$, x and $PID_{i}$ to compute

$C_{1}^{*}=h\left(PID_{i}∥ID_{CS}∥x\right)$,

$r_{U}=D_{1}⊕h\left(PID_{i}∥ID_{CS}∥x\right)$,

$ID_{i}=D_{2}⊕h\left(r_{U}∥PID_{i}∥ID_{CS}\right)$, and

$PID_{i}^{new}=D_{3}⊕h\left(ID_{i}∥x\right)⊕h\left(r_{U}∥ID_{i}\right)$.

Step 4: After CS verifies the authenticity of $ID_{i}$ and $D_{4}$, CS computes and sends

$D_{12}=h\left(PID_{i}^{new}∥ID_{cs}∥x\right)⊕h\left(r_{U}∥PID_{i}^{new}\right)$,

$D_{13}=h\left(PID_{i}^{new}∥r_{U}∥PID_{i}\right)⊕\left(r_{S}⊕r_{SC}\right)$, and

$D_{14}=h\left(SK_{CS}∥D_{12}∥D_{13}∥h\left(ID_{i}∥x\right)\right)$.

Step 5: Upon receiving the authentication response message $M_{8}$, the attacker computes

$\left(r_{S}⊕r_{SC}\right)=D_{13}⊕h\left(PID_{i}^{new}∥r_{U}∥PID_{i}\right)$ and

$SK_{U}=h\left(r_{U}⊕r_{S}⊕r_{CS}\right)$.

As a consequence, the attacker can launch the replay attack successfully.

User Impersonation Attack

The attacker can compute a valid authentication request message which contains the correct parameters to be authenticated by CS. The description of the attack is presented below:

Step 1: The attacker knows $ID_{CS}$, x and PID. Thus, he/she computes

$C_{1}^{*}=h\left(PID_{i}∥ID_{CS}∥x\right)$.

Step 2: The attacker recovers $r_{U}$ from $D_{1}$ computing

$r_{U}=D_{1}⊕h\left(PID_{i}∥ID_{CS}∥x\right)$.

Step 3: The attacker has enough information to recover sensitive data as follows

$ID_{i}=D_{2}⊕h\left(r_{U}∥PID_{i}∥ID_{CS}\right)$ and

$PID_{i}^{new}=D_{3}⊕h\left(ID_{i}∥x\right)⊕h\left(r_{U}∥ID_{i}\right)$.

Step 4: From this point, the attacker computes a fake authentication request message as follows:

$D_{1}^{fake}=C_{1}^{*}⊕r_{U}^{fake}$,

$D_{2}^{fake}=h\left(PID_{i}^{new}∥ID_{CS}∥r_{U}^{fake}\right)⊕ID_{i}$,

$D_{3}^{fake}=C_{2}^{*}⊕PID_{i}^{fake}⊕h\left(r_{U}^{fake}∥ID_{i}\right)$, and

$D_{4}^{fake}=h\left(ID_{i}∥PID_{i}^{new}∥PID_{i}^{fake}∥r_{U}^{fake}∥D_{3}^{fake}\right)$.

Step 5: The attacker sends the fake authentication request message $M_{5}^{fake}=\left\{PID_{i}^{new},\;D_{1}^{fake},D_{2}^{fake},\;D_{3}^{fake},D_{4}^{fake}\right\}$ to $S_{j}$. After $S_{j}$ finalizes the process, $S_{j}$ sends the authentication request message $M_{6}=\left\{PID_{i}^{new},\;D_{1}^{fake},D_{2}^{fake},\;D_{3}^{fake},D_{4}^{fake},PSID_{j},D_{5},D_{6},D_{7},D_{8}\right\}$ to CS. Upon receiving the authentication request message $M_{6}$, CS carries out the verification process of $D_{4}^{fake}$ computing $h\left(ID_{i}∥PID_{i}^{new}∥PID_{i}^{fake}∥r_{U}^{fake}∥D_{3}^{fake}\right)$ and verifying $D_{4}^{fake}\;?=h\left(ID_{i}∥PID_{i}^{new}∥PID_{i}^{fake}∥r_{U}^{fake}∥D_{3}^{fake}\right)$. It is obvious that, $D_{4}^{fake}$ will pass the verification process because it contains the original $ID_{i}$ and last $PID_{i}^{new}$.

### 2.4. Security Drawbacks

In this subsection, we expose the absence of security requirements in the scheme of Zhou et al. [30]. The security analysis was conducted based on [5,6,15]. From these references, we initialized the security analysis.

#### 2.4.1. Fails to Provide Mutual Authentication

The scheme proposed by Zhou et al. does not provide mutual authentication. The CS verifies the identity of $U_{i}$ and $S_{j}$ during the third step of the authentication phase; however, neither the user nor server verifies the identity of each other. Moreover, the authentication request messages $M_{5}$ and $M_{6}$ do not contain information which establishes a relationship between $U_{i}$ and $S_{j}$, as evidence to the attempt of connection.

#### 2.4.2. Fails to Protect Secret Key

In the scheme proposed by Zhou et al., the CS uses the same secret key (x) to register users and servers. Moreover, the secret key is hidden by means of $C_{1}^{*}=h\left(PID_{i}∥ID_{CS}∥x\right)$ and $C_{2}^{*}=h\left(ID_{i}∥x\right)$; however an attacker can recover it for three reasons.

The first reason is related with the fact that CS uses the same secret key to register each user and each server, increasing the possibility of finding the correct value of x. The second reason is related with the fact that an attacker knows $ID_{CS}$ and $PID_{i}$; this means that, each user knows two of three security parameters, decreasing the entropy to find x, in polynomial time. Finally, the low execution time of the hash function makes possible to find the secret key in polynomial time, in specific, using $C_{2}^{*}=h\left(ID_{i}∥x\right)$ because the attacker knows $ID_{i}$.

## 3. Proposed Scheme

In this section, we present our new version of a lightweight IoT-based authentication scheme in cloud computing circumstances. The scheme includes mutual authentication and key agreement to provide strong security for accessing any server of the cloud. The scheme consists of the following phases:

Registration is the process through CS creates the security parameters of each member of the system. This phase is mandatory for users and servers. The communication among participants is through a secure channel avoiding eavesdropper.

Login is the process through $U_{i}$ gets access to security parameters stored in his/her $SC_{U}$. $U_{i}$ needs to insert his/her $SC_{U}$, and inputs $ID_{i}$ and $PW_{i}$. Then, $SC_{U}$ computes and verifies the legitimacy of $U_{i}$. If the verification process is correct, $U_{i}$ sends the authentication request message to $S_{j}$ through an open communication channel.

Authentication is the processes by CS carries out the validation process of $U_{i}$ and $S_{j}$. Moreover, CS verifies that both entities want to establish a secure communication.

Key agreement is the process by CS computes the session key for $U_{i}$ and $S_{j}$. The session key is unique.

Mutual authentication is the process through $U_{i}$ and $S_{j}$ verifies the legitimacy of each other. In this case, $U_{i}$ sends a challenge to $S_{j}$. If the response is correct, $U_{i}$ knows that $S_{j}$ is a member of the system.

Table 1 summarizes the notations used throughout our proposal.

### 3.1. Registration Phase

This phase includes user registration and server registration.

#### 3.1.1. User Registration Sub-Phase

**Figure 2:**
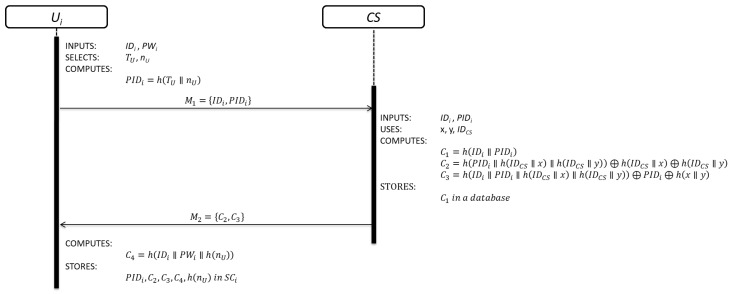
User registration sub-phase.

This sub-phase is initialized by $U_{i}$ when wants to be part of the system. The details of each step are shown in Figure 2.

Step 1: $U_{i}$ inserts his/her $SC_{i}$ into a device and keys his/her $ID_{i}$ and $PW_{i}$. Then, $SC_{i}$ generates a random nonce $\left(n_{U}\right)$, obtains the current timestamp value $\left(T_{U}\right)$ and computes:

(1) $$PID_{i}=h\left(T_{U}∥n_{U}\right)$$

$$U_{i}\Rightarrow CS:M_{1}=\left\{ID_{i},PID_{i}\right\}$$

Equation (1) is used to compute the pseudo-identity of each user.

Step 2: After receiving the registration request message $M_{1}$, CS verifies the validity of $ID_{i}$. If $ID_{i}$ is valid, CS computes:

(2) $$C_{1}=h\left(ID_{i}∥PID_{i}\right)$$

(3) $$C_{2}=h\left(PID_{i}∥h\left(ID_{CS}∥x\right)∥h\left(ID_{CS}∥y\right)\right)⊕h\left(ID_{CS}∥x\right)⊕h\left(ID_{CS}∥y\right)$$

(4) $$C_{3}=h\left(ID_{i}∥PID_{i}∥h\left(ID_{CS}∥x\right)∥h\left(ID_{CS}∥y\right)\right)⊕PID_{i}⊕h\left(x∥y\right)$$

$$STORES\;C_{1}\;in\;a\;database$$

$$CS\Rightarrow U_{i}:\;M_{2}=\left\{C_{2},\;C_{3}\right\}$$

CS registers $U_{i}$ by Equation (2). Equations (3) and (4) are security parameters for future authentication purpose.

Step 3: Upon receiving the registration response message $M_{2}$, $SC_{i}$ computes:

(5) $$C_{4}=h\left(ID_{i}∥PW_{i}∥h\left(n_{U}\right)\right)$$

$$STORES\;PID_{i},C_{2},C_{3},C_{4},h\left(n_{U}\right)\;in\;SC_{i}$$

$SC_{i}$ computes the local user authentication parameter using Equation (5) and stores all the security parameters received from CS. The user registration sub-phase is over.

#### 3.1.2. Cloud Server Registration

**Figure 3:**
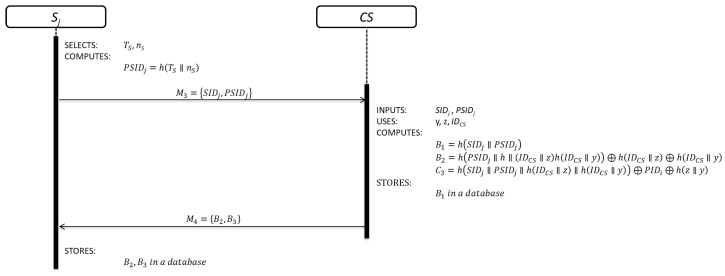
Server registration sub-phase.

This sub-phase is initialized by each $S_{j}$ in order to be part of the system. The details of each step are shown in Figure 3.

Step 1: $S_{j}$ generates a random nonce $n_{S}$, obtains the current timestamp value $T_{S}$ and computes:

(6) $$PSID_{j}=h\left(T_{S}∥n_{S}\right)$$

$$S_{j}\Rightarrow CS:M_{3}=\left\{SID_{j},PSID_{j}\right\}$$

Equation (6) is used to compute the pseudo-identity of each server.

Step 2: After receiving the registration request message $M_{3}$, CS verifies the validity of $SID_{j}$. If $SID_{j}$ is correct, CS computes:

(7) $$B_{1}=h\left(SID_{j}∥PSID_{j}\right)$$

(8) $$B_{2}=h\left(PSID_{j}∥h\left(ID_{CS}∥z\right)∥h\left(ID_{CS}∥y\right)\right)⊕h\left(ID_{CS}∥z\right)⊕h\left(ID_{CS}∥y\right)$$

(9) $$B_{3}=h\left(SID_{j}∥PSID_{j}∥h\left(ID_{CS}∥z\right)∥h\left(ID_{CS}∥y\right)\right)⊕PSID_{j}⊕h\left(z∥y\right)$$

$$STORES\;B_{1}\;in\;a\;database$$

$$CS\Rightarrow S_{j}:M_{4}=\left\{B_{2},B_{3}\right\}$$

CS registers $S_{j}$ by Equation (7). Equations (8) and (9) are security parameters for future authentication purpose.

Step 3: After receiving the registration response message $M_{4}$, $S_{j}$ stores $B_{2}$ and $B_{3}$.

### 3.2. Login Phase

**Figure 4:**
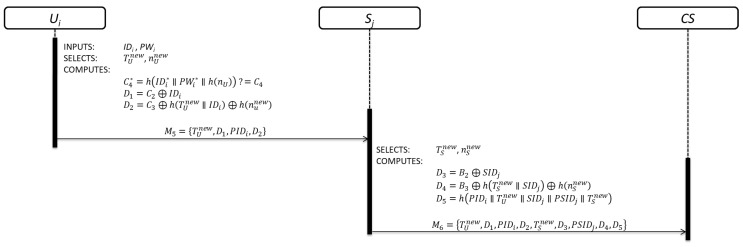
Login phase.

Once $U_{i}$ was registered, $U_{i}$ can connect to any server of the cloud by initiating the login phase. The details are shown in Figure 4.

Step 1: In order to start the authentication phase, $U_{i}$ must first complete the login phase. Firstly, $U_{i}$ inserts $SC_{i}$ and keys his/her $ID_{i}^{*}$ and $PW_{i}^{*}$.

Step 2: Then, $SC_{i}$ computes and checks:

(10) $$C_{4}^{*}=h\left(ID_{i}^{*}∥PW_{i}^{*}∥h\left(n_{U}\right)\right)\;?=C_{4}$$

Equation (10) is used to verify the legitimacy of $U_{i}$ by $SC_{i}$ for getting access to security parameters provided by $S_{j}$.

Step 3: If the verification process is correct, $SC_{i}$ generates a random nonce $n_{U}^{new}$, obtains the current timestamp value $T_{U}^{new}$ and computes:

(11) $$D_{1}=C_{2}⊕ID_{i}$$

(12) $$D_{2}=C_{3}⊕h\left(T_{U}^{new}∥ID_{i}\right)⊕h\left(n_{u}^{new}\right)$$

$$U_{i}\to S_{j}:M_{5}=\left\{T_{U}^{new},D_{1},PID_{i},D_{2}\right\}$$

Equations (11) and (12) contain $U_{i}$´s information which will be used by CS to verify its legitimacy. Finally, $U_{i}$ sends the user authentication request message $M_{5}$ to $S_{j}$ through an open communication channel.

Step 4: After receiving the user authentication request message $M_{5}$, $S_{j}$ generates a random nonce $n_{S}^{new}$, obtains the current timestamp value $T_{S}^{new}$ and computes:

(13) $$D_{3}=B_{2}⊕SID_{j}$$

(14) $$D_{4}=B_{3}⊕h\left(T_{S}^{new}∥SID_{j}\right)⊕h\left(n_{S}^{new}\right)$$

(15) $$D_{5}=h\left(PID_{i}∥T_{U}^{new}∥SID_{j}∥PSID_{j}∥T_{S}^{new}\right)$$

$$S_{j}\to CS:M_{6}=\left\{T_{U}^{new},D_{1},PID_{i},D_{2},T_{S}^{new},D_{3},PSID_{j},D_{4},D_{5}\right\}$$

Equations (13) and (14) contain $S_{j}$´s information which will be used by CS to verify its legitimacy. Equation (15) contains information about $U_{i}$ and $S_{j}$ as evidence of its connection attempt. Finally, $S_{j}$ sends the authentication request message $M_{6}$ to CS through an open communication channel.

### 3.3. Authentication Phase

**Figure 5:**
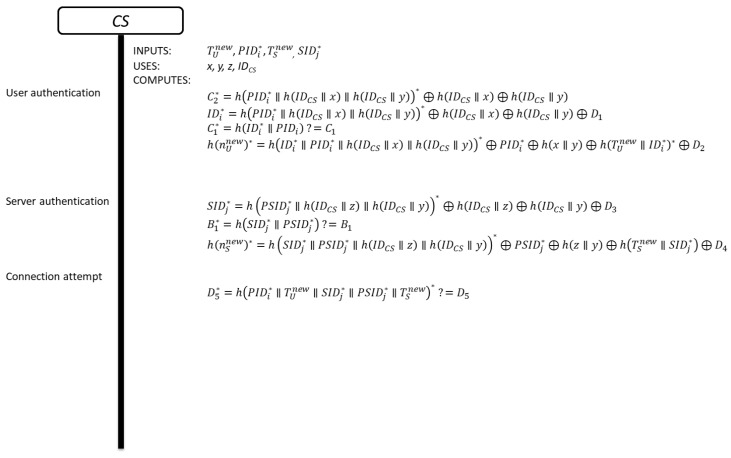
User authentication, server authentication and evidence of connection attempt sub-phases.

This phase is divided in three sub-phases. The details of user authentication, server authentication and evidence of connection attempt are shown in Figure 5.

#### 3.3.1. User Authentication

Step 1: Upon receiving the authentication request message $M_{6}$, CS checks the freshness of the message by means of $T_{U}^{new}$. If the verification process is positive, CS computes:

$$C_{2}^{*}=h{\left(PID_{i}^{*}∥h\left(ID_{CS}∥x\right)∥h\left(ID_{CS}∥y\right)\right)}^{*}⊕h\left(ID_{CS}∥x\right)⊕h\left(ID_{CS}∥y\right)$$

where $PID_{i}^{*}$ is the pseudo-identity of $U_{i}$ contained in message $M_{2}$. CS computed $C_{2}^{*}$ using $PID_{i}^{*}$ and Equation (3).

Step 2: CS verifies the legitimacy of $U_{i}$ by means of $C_{1}$ as follows:

(16) $$D_{1}=C_{2}^{*}⊕ID_{i}\;D_{1}=h{\left(PID_{i}^{*}∥h\left(ID_{CS}∥x\right)∥h\left(ID_{CS}∥y\right)\right)}^{*}⊕h\left(ID_{CS}∥x\right)⊕h\left(ID_{CS}∥y\right)⊕ID_{i}\;ID_{i}^{*}=h{\left(PID_{i}^{*}∥h\left(ID_{CS}∥x\right)∥h\left(ID_{CS}∥y\right)\right)}^{*}⊕h\left(ID_{CS}∥x\right)⊕h\left(ID_{CS}∥y\right)⊕D_{1}$$

(17) $$C_{1}^{*}=h\left(ID_{i}^{*}∥PID_{i}\right)\;?=C_{1}$$

Equation (16) is used to recover $ID_{i}^{*}$ from $D_{1}$. Equation (17) is used to verify the legitimacy of $U_{i}$ using $ID_{i}^{*}$ and $C_{1}$. If the verification process is correct, CS continues with the next step; otherwise, CS finalizes the process.

Step 3: After verifying the legitimacy of $U_{i}$, CS recovers $h\left(n_{U}^{new}\right)$ as follows:

(18) $$h{\left(n_{U}^{new}\right)}^{*}=h{\left(ID_{i}^{*}∥PID_{i}^{*}∥h\left(ID_{CS}∥x\right)∥h\left(ID_{CS}∥y\right)\right)}^{*}⊕PID_{i}^{*}⊕h\left(x∥y\right)⊕h{\left(T_{U}^{new}∥ID_{i}^{*}\right)}^{*}⊕D_{2}$$

Equation (18) is used to recover $h{\left(n_{U}^{new}\right)}^{*}$ from $D_{2}$.

Step 4: CS computes $C_{1}^{new}$ with $T_{U}^{new}$ and $h{\left(n_{U}^{new}\right)}^{*}$ using Equation (19). Then, CS updates $C_{1}$ in the database:

(19) $$C_{1}^{new}=h\left(ID_{i}∥h\left(T_{U}^{new}∥C_{1}∥h\left(n_{U}^{new}\right)\right)\right)$$

#### 3.3.2. Server Authentication

Step 1: Upon finalizing the user authentication process, CS checks the freshness of the message by means of $T_{S}^{new}$.

Step 2: If the verification process is positive, CS verifies the legitimacy of $S_{j}$ as follows:

(20) $$D_{3}=B_{2}⊕SID_{j}\;B_{2}=h{\left(PSID_{j}^{*}∥h\left(ID_{CS}∥z\right)∥h\left(ID_{CS}∥y\right)\right)}^{*}⊕h\left(ID_{CS}∥z\right)⊕h\left(ID_{CS}∥y\right)⊕SID_{j}\;SID_{j}^{*}=h{\left(PSID_{j}^{*}∥h\left(ID_{CS}∥z\right)∥h\left(ID_{CS}∥y\right)\right)}^{*}⊕h\left(ID_{CS}∥z\right)⊕h\left(ID_{CS}∥y\right)⊕D_{3}$$

(21) $$B_{1}^{*}=h\left(SID_{j}^{*}∥PSID_{j}^{*}\right)\;?=B_{1}$$

Equation (20) is used to recover $SID_{j}^{*}$ from $D_{3}$, using $PSID_{j}^{*}$. Then, CS computes Equation (21) to obtain $B_{1}^{*}$ and compares it with $B_{1}$. If the verification process is correct, CS continues with the process, otherwise, CS finalizes the process.

Step 3: After verifying the legitimacy of $S_{j}$, CS recover $h\left(n_{S}^{new}\right)$ as follows:

(22) $$h{\left(n_{S}^{new}\right)}^{*}=h{\left(SID_{j}^{*}∥PSID_{j}^{*}∥h\left(ID_{CS}∥z\right)∥h\left(ID_{CS}∥y\right)\right)}^{*}⊕PSID_{j}^{*}⊕h\left(z∥y\right)⊕h\left(T_{S}^{new}∥SID_{j}^{*}\right)⊕D_{4}$$

Finally, Equation (22) is used to recover $h{\left(n_{S}^{new}\right)}^{*}$ from $D_{4}$.

#### 3.3.3. Evidence of Connection Attempt

Step 1: CS corroborates that $U_{i}$ wants to establish a connection with $S_{j}$ as follows:

(23) $$D_{5}^{*}=h{\left(PID_{i}^{*}∥T_{U}^{new}∥SID_{j}^{*}∥PSID_{j}^{*}∥T_{S}^{new}\right)}^{*}\;?=D_{5}$$

in this case, CS has evidence of the connection attempt between $U_{i}$ and $S_{j}$. It is important to note that, Equation (23) requires the fresh timestamp from $U_{i}$ and $S_{j}$. Moreover, $D_{5}$ contains $PID_{i}$, $SID_{j}$ and $PSID_{j}$ which demonstrate the interest of the two entities for establishing a secure communication.

### 3.4. Key Agreement Phase

**Figure 6:**
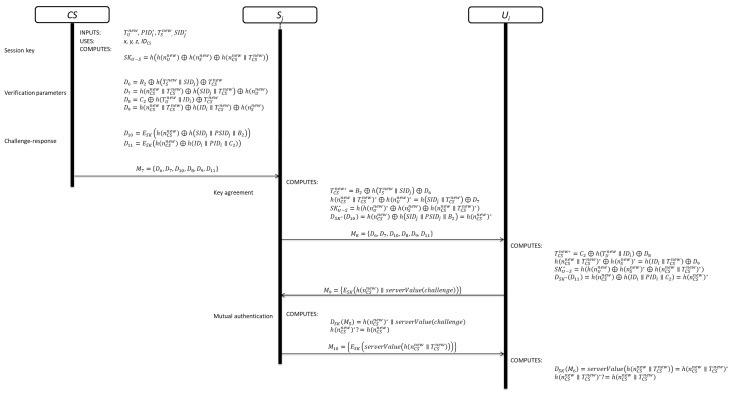
Key agreement and mutual authentication phases.

This phase is divided in three sub-phases. The details of each phase are shown in Figure 6.

#### 3.4.1. Session Key Creation

In this sub-phase, CS computes the session key between $U_{i}$ and $S_{j}$ as follows:

Step 1: CS generates a random nonce $n_{CS}^{new}$ and computes the session key $SK_{U-S}$ as follows:

(24) $$SK_{U-S}=h\left(h\left(n_{U}^{new}\right)⊕h\left(n_{S}^{new}\right)⊕h\left(n_{CS}^{new}∥T_{CS}^{new}\right)\right)$$

Equation (24) is used to compute the session key. The session key contains security parameters generated by $U_{i}$, $S_{j}$ and CS which represents the relationship among all the participants.

Step 2: CS computes the verification parameters for $S_{j}$ and $U_{i}$ as follows:

(25) $$D_{6}=B_{2}⊕h\left(T_{S}^{new}∥SID_{j}\right)⊕T_{CS}^{new}$$

(26) $$D_{7}=h\left(n_{CS}^{new}∥T_{CS}^{new}\right)⊕h\left(SID_{j}∥T_{CS}^{new}\right)⊕h\left(n_{U}^{new}\right)$$

(27) $$D_{8}=C_{2}⊕h\left(T_{U}^{new}∥ID_{i}\right)⊕T_{CS}^{new}$$

(28) $$D_{9}=h\left(n_{CS}^{new}∥T_{CS}^{new}\right)⊕h\left(ID_{i}∥T_{CS}^{new}\right)⊕h\left(n_{S}^{new}\right)$$

where $D_{6}$ and $D_{7}$ are for $S_{j}$, while $D_{8}$ and $D_{9}$ are for $U_{i}$. Equations (25) to (28) contain information generated by CS for computing the session key.

Step 3: CS computes the challenge-response message for $S_{j}$ and $U_{i}$ as follows:

(29) $$D_{10}=E_{SK}\left(h\left(n_{CS}^{new}\right)⊕h\left(SID_{j}∥PSID_{j}∥B_{2}\right)\right)$$

(30) $$D_{11}=E_{SK}\left(h\left(n_{CS}^{new}\right)⊕h\left(ID_{i}∥PID_{i}∥C_{2}\right)\right)$$

$$CS\to S_{j}:M_{7}=\left\{D_{6},D_{7},D_{10},D_{8},D_{9},D_{11}\right\}$$

CS computed the challenge-response message for each entity using the session key; this means that, a legitimate participant can recover the security parameters to construct the session key. Finally, CS sends the authentication response message $M_{7}$ to $S_{j}$ through an open communication channel.

#### 3.4.2. Server Session Key

Step 1: After receiving $M_{7}$, $S_{j}$ computes and verifies the freshness of $M_{7}$:

(31) $$T_{CS}^{new*}=B_{2}⊕h\left(T_{S}^{new}∥SID_{j}\right)⊕D_{6}$$

Equation (31) is used to extract $T_{CS}^{new*}$ from $D_{6}$.

Step 2: If $M_{7}$ is fresh, $S_{j}$ computes:

(32) $$h{\left(n_{CS}^{new}∥T_{CS}^{new}\right)}^{*}⊕h{\left(n_{U}^{new}\right)}^{*}=h\left(SID_{j}∥T_{CS}^{new}\right)⊕D_{7}$$

$$SK_{U-S}^{*}=h\left(h{\left(n_{U}^{new}\right)}^{*}⊕h\left(n_{S}^{new}\right)⊕h{\left(n_{CS}^{new}∥T_{CS}^{new}\right)}^{*}\right)$$

(33) $$h{\left(n_{CS}^{new}\right)}^{*}=h\left(n_{CS}^{new}\right)⊕h\left(SID_{j}∥PSID_{j}∥B_{2}\right)=D_{SK^{*}}\left(D_{10}\right)$$

$$S_{j}\to U_{i}:M_{8}=\left\{D_{8},D_{9},D_{11}\right\}$$

at this point, $S_{j}$ knows the session key $\left(SK_{U-S}^{*}\right)$ and the value $h{\left(n_{CS}^{new}\right)}^{*}$. Finally, $S_{j}$ sends $M_{8}$ to $U_{i}$ through an open communication channel.

#### 3.4.3. User Session Key

Step 1: Upon receiving the user authentication response message $M_{8}$, $U_{i}$ computes and verifies the freshness of $M_{8}$:

(34) $$T_{CS}^{new*}=C_{2}⊕h\left(T_{U}^{new}∥ID_{i}\right)⊕D_{8}$$

Step 2: If $M_{8}$ is fresh, $U_{i}$ computes:

(35) $$h{\left(n_{CS}^{new}∥T_{CS}^{new}\right)}^{*}⊕h{\left(n_{S}^{new}\right)}^{*}=h\left(ID_{i}∥T_{CS}^{new}\right)⊕D_{9}$$

$$SK_{U-S}^{*}=h\left(h\left(n_{U}^{new}\right)⊕h{\left(n_{S}^{new}\right)}^{*}⊕h{\left(n_{CS}^{new}∥T_{CS}^{new}\right)}^{*}\right)$$

(36) $$D_{SK^{*}}\left(D_{11}\right)=h\left(n_{CS}^{new}\right)⊕h\left(ID_{i}∥PID_{i}∥C_{2}\right)=h{\left(n_{CS}^{new}\right)}^{*}$$

$U_{i}$ knows the session key and the value $h{\left(n_{CS}^{new}\right)}^{*}$.

### 3.5. Mutual Authentication

Step 1: $U_{i}$ sends the challenge message $M_{9}$ to $S_{j}$. $M_{9}$ contains $h\left(n_{CS}^{new}\right)$ as proof of his/her legitimacy and requests the response:

$$U_{i}\to S_{j}:M_{9}=\left\{E_{SK}\left(h\left(n_{CS}^{new}\right)∥serverValue\left(challenge\right)\right)\right\}$$

Step 2: Upon receiving the challenge message $M_{9}$, $S_{j}$ computes

(37) $$h{\left(n_{CS}^{new}\right)}^{*}∥serverValue\left(challenge\right)=D_{SK}\left(M_{9}\right)$$

(38) $$h{\left(n_{CS}^{new}\right)}^{*}?=h\left(n_{CS}^{new}\right)$$

$$S_{j}\to U_{i}:M_{10}=\left\{E_{SK}\left(serverValue\left(h\left(n_{CS}^{new}∥T_{CS}^{new}\right)⊕h\left(n_{U}^{new}\right)\right)\right)\right\}$$

$S_{j}$ knows that $U_{i}$ is the user who requested the user authentication by means of Equations (37) and (38). Then, $S_{j}$ sends the response to $U_{i}$.

Step 3: Upon receiving the message $M_{10}$, $U_{i}$ computes and verifies the legitimacy of $S_{j}$ as follows:

(39) $$h{\left(n_{CS}^{new}∥T_{CS}^{new}\right)}^{*}⊕h\left(n_{U}^{new}\right)=serverValue\left(h\left(n_{CS}^{new}∥T_{CS}^{new}\right)⊕h\left(n_{U}^{new}\right)\right)=D_{SK}\left(M_{10}\right)$$

(40) $$h{\left(n_{CS}^{new}∥T_{CS}^{new}\right)}^{*}?=h\left(n_{CS}^{new}∥T_{CS}^{new}\right)$$

finally, $U_{i}$ recovers the response from $M_{10}$ using Equation (39) and verifies the legitimacy of $S_{j}$ by means of Equation (40).

Step 4: $U_{i}$ replaces $PID_{i}$, $h\left(n_{U}\right)$, and $C_{4}=h\left(ID_{i}∥PW_{i}∥h\left(n_{U}\right)\right)$ with $PID_{i}^{new}=\left(h\left(T_{U}^{new}∥h\left(ID_{i}∥PID_{i}\right)∥n_{U}^{new}\right)\right)$, $h\left(n_{U}^{new}\right)$, and $C_{4}^{new}=h\left(ID_{i}∥PW_{i}∥h\left(n_{U}^{new}\right)\right)$, respectively.

### 3.6. Password Change Phase

When $U_{i}$ wants to change or to update his/her password, he/she needs to key his/her $ID_{i}$ and $PW_{i}$. Then, $SC_{i}$ computes Equation (10) to verify his/her legitimacy. If the verification process is correct, $U_{i}$ keys $PW_{i}^{new}$ and $SC_{i}$ computes Equation (40):

(41) $$C_{4}^{new}=h\left(ID_{i}∥PW_{i}^{new}∥h\left(n_{u}\right)\right)$$

$$UPDATES\;C_{4}$$

## 4. Security Analysis and Performance Evaluation

In this section, we carry out the security analysis and performance evaluation comparison of our proposal. The security analysis includes an informal cryptanalysis, security of session key and countermeasures to improve security. The performance evaluation includes computational- and communication-cost comparison with Zhou et al.’s, Amin et al.’s and Xue et al.’s schemes.

### 4.1. Informal Cryptanalysis

In this sub-section, we analyse the security of our proposal using informal security analysis.

#### 4.1.1. User Anonymity

In our scheme, $U_{i}$ sends $PID_{i}=h\left(T_{U}∥n_{U}\right)$ to $S_{j}$ instead of $ID_{i}$ in clear text. Moreover, $PID_{i}$ is updated after finalizing the user authentication sub-phase with $T_{U}^{new}$ and $n_{U}^{new}$, keeping the identity of each user anonym. In consequence, when the user sends the authentication request message to $S_{j}$, the $PID_{i}$ will be different. Furthermore, an attacker cannot recover $ID_{i}$ from $PID_{i}$, $D_{1}$, or $D_{2}$ without security parameters. Therefore, the scheme provides user anonymity.

#### 4.1.2. Off-line User Identity and Password Guessing Attack

In the case that a malicious user obtains $SC_{i}$, he/she can recover $PID_{i},C_{2},C_{3},C_{4},h\left(n_{U}\right)$ [33,34]. However, he/she cannot obtain sensitive data from $C_{2}$ because nobody knows *x*, *y*, *z*, and *ID_CS_*. From $C_{3}$ the attacker can extract $PID_{i}$ which it is stored in $SC_{i}$. In this case, he/she is not capable to extract sensitive data from $SC_{i}$.

#### 4.1.3. Privileged Insider Attack

In our scheme, security parameters are personalized using data from each user or cloud server, making more complex the possibility to know secret keys of CS. If a malicious user tries to extract x and y from $C_{2}$ or $C_{3}$, he/she needs to recover $h\left(ID_{CS}∥x\right)$ or $h\left(ID_{CS}∥y\right)$ from Equations (3) or (4):

$$C_{2}=h\left(PID_{i}∥h\left(ID_{CS}∥x\right)∥h\left(ID_{CS}∥y\right)\right)⊕h\left(ID_{CS}∥x\right)⊕h\left(ID_{CS}∥y\right)$$

$$C_{3}=h\left(ID_{i}∥PID_{i}∥h\left(ID_{CS}∥x\right)∥h\left(ID_{CS}∥y\right)\right)⊕PID_{i}⊕h\left(x∥y\right)$$

but the attacker only know $PID_{i}$. Moreover, CS does not include its $ID_{CS}$ in any messages or shares it in clear text. Thus, the scheme resists this attack.

#### 4.1.4. Impersonation Attack

In the case that an attacker obtains $PID_{i},C_{2},C_{3},C_{4},h\left(n_{U}\right)$ from $SC_{i}$ [33,34] and $M_{5}=\left\{T_{U}^{new},D_{1},PID_{i},D_{2}\right\}$ the attacker cannot create a valid authentication request message by any type of combination of the security parameters. In this case, the malicious user computes $D_{1}^{fake}=C_{2}⊕ID_{i}^{fake}$ using $ID_{i}^{fake}$ but he/she cannot compute a valid $PID_{i}$ and $C_{3}$ which are required to compute a valid $D_{2}$. In consequence, the attacker cannot impersonate a legal user.

#### 4.1.5. Replay Attack

In this attack, the malicious user needs to know previous authentication request message $M_{5}=\left\{T_{U}^{new},D_{1},PID_{i},D_{2}\right\}$. However, our scheme uses random nonce $\left(n_{U}\right)$ and timestamp $\left(T_{U}\right)$ to avoid replay attack. The control server verifies the freshness of the timestamp every time.

### 4.2. Security of Session Key

A key purpose of an authentication scheme is the establishment of a session key, so the session key should be protected against known-key security and forward secrecy [22].

#### 4.2.1. Known-key Security

In our scheme, CS computes new session key every time the authentication is correct. This means that, CS uses the new random nonce $\left(n_{U}^{new}\right)$, $\left(n_{S}^{new}\right)$ and $\left(n_{CS}^{new}\right)$, and its current timestamp $\left(T_{CS}^{new}\right)$ to compute a fresh session key, avoiding the compromise of previous session keys. If the attacker knows $M_{7}=\left\{D_{6},D_{7},D_{10},D_{8},D_{9},D_{11}\right\}$, he/she cannot compute a valid session key $\left(SK_{U-S}\right)$ without random nonce and CS’s timestamp. Even though the attacker knows past session key $\left(SK_{U-S}^{old}\right)$, he/she cannot compute the new session key by means of any type of combination.

#### 4.2.2. Forward Secrecy

In our scheme, CS computes the session key without the use of secret keys (x,y,z), avoiding compromise its security in case that an attacker knows the secret keys. Let us suppose that, an attacker knows (x,y,z), he/she cannot compute the correct session key because it does not contain the secret keys. Thus, the attacker cannot create a valid session key.

### 4.3. Countermeasures

#### 4.3.1. Local Protection against Malicious Users

In our scheme, $SC_{i}$ verifies the legitimacy of $U_{i}$ by means of Equation (10). This mean that $U_{i}$ must be authenticated by $SC_{i}$ before it computes the user authentication request message [22].

#### 4.3.2. Mutual Authentication

In our scheme, $U_{i}$ and $S_{j}$ verify that each other is a legitimate user in the system and want to establish a secure communication through Equations (37) to (40). In this case, $U_{i}$ sends a challenge to $S_{j}$ for carrying out the mutual authentication process. The response message contains $\left(n_{CS}^{new}∥T_{CS}^{new}\right)$ which represents the fresh of the communication. Moreover, $U_{i}$ knows the same value, thus avoiding a man-in-the-middle attack.

#### 4.3.3. Evidence of Connection Attempt

In our scheme, $S_{j}$ computes $D_{5}$, using Equation (15), which contains information from $U_{i}$ and $S_{j}$, making unique the value of $D_{5}$. Then, CS verifies the connection attempt between $U_{i}$ and $S_{j}$ by means of Equation (23). Moreover, CS computes the session key using information of $U_{i}$, $S_{j}$ and CS.

### 4.4. Security Comparison

This sub-section presents the security comparison of the proposed scheme with Zhou et al.’s scheme, Amin et al.’s scheme and Xue et al.’s schemes in terms of security properties. Table 2 lists comparative results.

According to Table 2, it is clear that previous works are vulnerable to different attacks and fails to provide mutual authentication between the server and the user. Moreover, previous works do not provide evidence of connection attempts. In consequence, our protocol resists very well-known attacks, provides evidences of connection attempts, mutual authentication and user anonymity.

### 4.5. Computational-cost Comparison

This sub-section presents the performance evaluation of the proposed scheme with Zhou et al.’s scheme, Amin et al.’s scheme and Xue et al.’s scheme in terms of execution-time. The evaluation of each scheme was based on the following considerations:

- *T_h_* represents a hash function.
- *T_S_* represents an encryption/decryption operation using AES algorithm.
- The execution time for *T_h_* is case 1: 0.00517 ms [30] and case 2: 0.0000328 ms [32].
- The execution time for *T_S_* is case1: 0.02148 ms [30] and case2: 0.0214385 ms [32].

Table 3 summarizes the operations carried out by $U_{i},\;S_{j}$ and CS during the registration, login and authentication phases. The execution time required by $U_{i},\;S_{j}$ and CS during each phase is shown in Table 4.

From Table 3, it is easy to see that our scheme requires more computational operations than previous works. However, it is necessary to compute the execution-time in a real scenario. For that reason, we used the execution-time described in [30] and [32] to compare the performance of each scheme. The execution-time results are shown in Table 4, and it is clear that the execution time depends on: (a) the characteristics of each device, (b) the cryptography libraries and (c) the computational load. In our scheme, CS is the participant which computes more computational operations because we assume that has more resources than $U_{i}$ and $S_{j}$. Moreover, $U_{i}$ computes more computational operations than $S_{j}$ because we assume that $U_{i}$ will request few connections per day; however, $S_{j}$ will receive many request for connection, considering a high volume of users it is necessary that $S_{j}$ computes few computational operations. According with the results summarized in Table 4, the scheme with les computational operations was proposed by Amin et al. [1].

In fact, the execution time of each device must be considered as show in Table 4. The computational operations evaluated using case 2 gives better results than case 1. Under this situation, our scheme requires 40% less time which represents an acceptable performance, less than 0.1862 ms [30].

**Figure 7:**
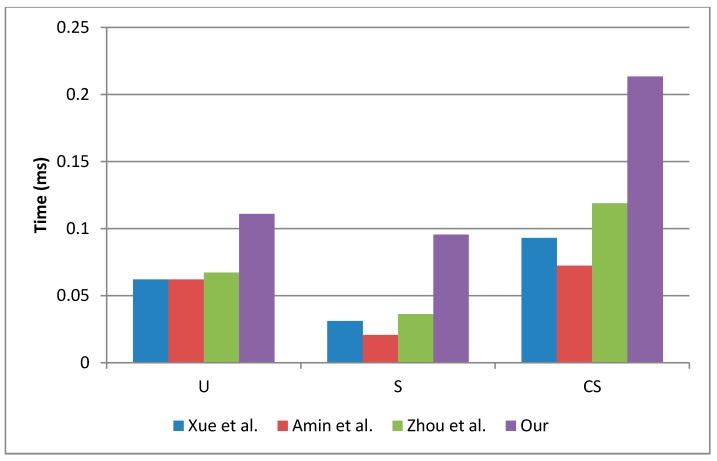
Computational-cost comparison by participant.

Figure 7 shows the computational-cost comparison by participant and scheme. In this case, we used the execution-time of the case 1. The main difference between our scheme and previous works is the inclusion of symmetric operations during the authentication phase. The symmetric operations are used to provide mutual authentication between $U_{i}$ and $S_{j}$, increasing the security of the proposed scheme.

### 4.6. Communication-Cost Comparison

In this sub-section, we compare the communication-cost of our scheme with Zhou et al.’s scheme, Amin et al.’s scheme and Xue et al.’s scheme in terms of message length. For conventional comparison, we assume two bit length cases:

- Case 1: any identity, password, pseudo-identity, timestamp, random nonce, and hash output are 128 bits.
- Case 2: any identity, password, pseudo-identity, timestamp, random nonce, and hash output are 256 bits.
- The block length of the symmetric encryption is 128 bits.

Table 5 summarizes the message length by each entity during the scheme.

From Table 5, we see very clearly that our scheme requires the same message length as Zhou et al.’s scheme, 4352 bits or 8704 bits. However, our scheme provides mutual authentication and evidence of connection attempt, which requires more information to share among participants. If we pay attention phase by phase, our scheme requires less message length during the registration phase than Zhou et al.’s scheme, making our proposal more efficient. In fact, our proposal requires less message length in the login phase than previous works, making it more efficient. After achieving the performance evaluation of the proposed scheme, it is possible to confirm that the proposal has good performance.

## 5. Conclusions

In this paper, we demonstrated that Zhou et al.’s scheme is not secure against insider, replay and user impersonation attacks. Moreover, we found security drawbacks which make the scheme proposed by Zhou et al. insecure for IoT in cloud computing circumstances. As a consequence, we propose a new scheme to remedy the security vulnerabilities and drawbacks of Zhou et al.’s scheme.

The new scheme achieves mutual authentication and key agreement, providing secure access to cloud servers. Moreover, the proposal keeps the user identity anonymous against eavesdroppers, provides security for the session key and includes a challenge-response method. In addition, the new scheme includes a sub-phase called evidence connection attempt which proves to the control server any connection attempt between a user and a server.

Furthermore, our performance evaluation demonstrates that our scheme does not require high computational power or several messages to achieve security requirements. On the contrary, the proposed scheme requires less communication-cost than Zhou et al.’s, Amin et al.’s and Xue et al.’s schemes in the registration and login phases, and the computational-cost is acceptable considering the security characteristics included in the scheme. Thus, the scheme meets the security requirements for a secure IoT-based authentication scheme in cloud computing circumstance, enhancing security of previous works.

## Figures and Tables

**Table 1:** Notations of the proposed scheme.

Symbol	Description
$U_{i}$	User
$S_{j}$	Cloud server
CS	Control server
$SC_{i}$	Smart card of $U_{i}$
$ID_{i},\;SID_{j},\;ID_{CS}$	Identity of $U_{i}$, $S_{j}$, CS, respectively
$PID_{i},\;PSID_{j}$	Pseudo-identity of $U_{i}$, $S_{j}$, respectively
$PW_{i}$	Password of $U_{i}$
x, y, z	Secret keys of CS. Secret keys are long integers
$n_{U},\;n_{S},\;n_{CS}$	Random nonce of $U_{i}$, $S_{j}$, CS, respectively
$T_{U},\;T_{S},\;T_{CS}$	Timestamp of $U_{i}$, $S_{j}$, CS, respectively
$SK_{U-S}$	Session key between $U_{i}$ and $S_{j}$
$E_{SK}\left(\cdot \right)/D_{SK}\left(\cdot \right)$	Symmetric encryption/decryption using $SK_{U-S}$
h(·)	Collision free one-way hash function
⊕	Exclusive-OR operation
∥	Concatenation operation
⇒	Secure communication channel
→	Open communication channel

**Table 2:** Security comparison.

Security Property	Xue et al.	Amin et al.	Zhou et al.	Our Scheme
Provide evidence of connection attempt	fails	fails	fails	success
Provide mutual authentication	fails	fails	fails	success
Provide user anonymity	fails			success
Resist impersonation attack	fails	fails	fails	success
Resist off-line user identity/password attack	fails			success
Resist privileged-insider attack	fails	fails		success
Resist replay attack			fails	success

**Table 3:** Performance comparison.

Phase		Xue et al.	Amin et al.	Zhou et al.	Our Scheme
Registration	$U_{i}$	$3T_{h}$	$3T_{h}$	$3T_{h}$	$2T_{h}$
	$S_{j}$	$0T_{h}$	$0T_{h}$	$0T_{h}$	$1T_{h}$
	CS	$4T_{h}$	$4T_{h}$	$4T_{h}$	$12T_{h}$
Login	$U_{i}$	$6T_{h}$	$6T_{h}$	$6T_{h}$	$3T_{h}$
	$S_{j}$	$3T_{h}$	$1T_{h}$	$3T_{h}$	$3T_{h}$
	CS	$0T_{h}$	$0T_{h}$	$0T_{h}$	$0T_{h}$
Authentication	$U_{i}$	$3T_{h}$	$3T_{h}$	$4T_{h}$	$4T_{h}+3T_{s}$
	$S_{j}$	$3T_{h}$	$3T_{h}$	$4T_{h}$	$2T_{h}+3T_{s}$
	CS	$14T_{h}$	$10T_{h}$	$19T_{h}$	$21T_{h}+2T_{s}$
Total		$36T_{h}$	$30T_{h}$	$43T_{h}$	$48T_{h}+8T_{s}$

**Table 4:** Execution-time by participant.

		Xue et al.			Amin et al.			Zhou et al.			Our		
		Op	Case 1	Case 2	Op	Case 1	Case 2	Op	Case 1	Case 2	Op	Case 1	Case 2
R	$U_{i}$	3Th	0.01551	0.0000984	3Th	0.01551	0.0000984	3TH	0.01551	0.0000984	2Th	0.01034	0.0000656
	$S_{j}$	0Th	0	0	0Th	0	0	0TH	0	0	1Th	0.00517	0.0000328
	CS	4Th	0.02068	0.0001312	4Th	0.02068	0.0001312	4TH	0.02068	0.0001312	12Th	0.06204	0.0003936
L	$U_{i}$	6Th	0.03102	0.0001968	6Th	0.03102	0.0001968	6Th	0.03102	0.0001968	3Th	0.01551	0.0000984
	$S_{j}$	3Th	0.01551	0.0000984	1Th	0.00517	0.0000328	3Th	0.01551	0.0000984	3Th	0.01551	0.0000984
	CS	0Th	0	0	0Th	0	0	0Th	0	0	0Th	0	0
A	$U_{i}$	3Th	0.01551	0.0000984	3Th	0.01551	0.0000984	4Th	0.02068	0.0001312	4Th + 3Ts	0.08512	0.0644467
	$S_{j}$	3Th	0.01551	0.0000984	3Th	0.01551	0.0000984	4Th	0.02068	0.0001312	2Th + 3Ts	0.07478	0.0643811
	CS	14Th	0.07238	0.0004592	10Th	0.0517	0.000328	19Th	0.09823	0.0006232	21Th + 2Ts	0.15153	0.0435658
Total		36Th	0.18612	0.0011808	30Th	0.1551	0.000984	43Th	0.22231	0.0014104	48Th + 8Ts	0.420000	0.173082

*R = Registration phase, L = Login phase, A = Authentication phase, Op = Number of operations.

**Table 5:** Communication cost comparison.

		Xue et al.			Amin et al.			Zhou et al.			Our Scheme		
		Length	Case 1	Case 2	Length	Case 1	Case 2	Length	Case 1	Case 2	Length	Case 1	Case 2
R	$U_{i}$	3	384	768	2	256	512	2	256	512	2	256	512
	$S_{j}$	2	256	512	2	256	512	2	256	512	2	256	512
	CS	2	256	512	3	384	768	6	768	1536	4	512	1024
	ST		896	1792		896	1792		1280	2560		1024	2048
L	$U_{i}$	6	768	1536	5	640	1280	5	640	1280	4	512	1024
	$S_{j}$	11	1408	2816	9	1152	2304	10	1280	2560	9	1152	2304
	CS	0	0	0	0	0	0	0	0	0	0	0	0
	ST		2176	4352		1792	3584		1920	3840		1664	3328
A	$U_{i}$	0	0	0	0	0	0	0	0	0	2	256	512
	$S_{j}$	2	256	512	2	256	512	3	384	768	5	640	1280
	CS	4	512	1024	4	512	1024	6	768	1536	6	768	1536
	ST		768	1536		768	1536		1152	2304		1664	3328
	T	30	3840	7680	27	3456	6912	34	4352	8704	34	4352	8704

*R = Registration phase, L = Login phase, A = Authentication phase, ST = Subtotal, T = Total.
